# Effects of retrograde reperfusion on the intraoperative internal environment and hemodynamics in classic orthotopic liver transplantation

**DOI:** 10.1186/s12893-018-0441-0

**Published:** 2018-12-12

**Authors:** Chongwei Yang, Lei Huang, Xinyu Li, Jiye Zhu, Xisheng Leng

**Affiliations:** 1 Department of Hepatobiliary Surgery, Peking University People’s Hospital, Xizhimen, Beijing, 100044 China; 2 Department of Hepatobiliary Surgery, The Central Hospital of Wuhan, Tongji Medical College, Huazhong University of Science and Technology, Wuhan, 430014 China

**Keywords:** Hemodynamics, Internal environment, Orthotopic liver transplantation, Postreperfusion syndrome, Retrograde reperfusion

## Abstract

**Background:**

To investigate the effects of retrograde reperfusion on the intraoperative internal environment and hemodynamics in classic orthotopic liver transplantation (OLT).

**Methods:**

Thirty patients were undergone classic OLT using retrograde reperfusion in our center. Blood sampling was done at different time points including: Before blood venting via the portal vein (PV), 10 mL of blood was collected from the inferior vena cava (T_0_); During retrograde reperfusion through the inferior vena cava (IVC), 10 mL of blood was collected when the volume of blood venting reached 10 mL (T_1_), 100 mL (T_2_), and 200 mL (T_3_), respectively. 5 mL of blood was analyzed using a NOVA-f–type Blood Gas Analyzer. The remaining 5 mL was measured to determine the level of IL-1β using an enzyme-linked immunosobent assay.

**Results:**

All operations were completed successfully, and postreperfusion syndrome (PRS) occurred in 6 patients (20%). The most notable findings were significant changes at T_1_, T_2_ and T_3_, including pH value, PvO_2_, SvO_2_, BEecf, HCO_3_^−^, Lac, K^+^, Ca^2+^ and IL-1β, compared with T_0_ (*P* < 0.05). Yet their levels at T_3_ were not back to the level at T_0_ (*P* < 0.05).

**Conclusion:**

This retrograde perfusion could eliminate some harmful metabolites inside the donor liver in time and reduce acid-base and electrolyte disorders as well as drastic hemodynamic fluctuations after recirculation during classic OLT.

## Background

Since Starzl et al. [1] performed the first orthotopic liver transplantation (OLT) for a pediatric patient with congenital biliary atresia in 1963, the OLT had been the most effective therapeutic approach for the treatment of end-stage liver diseases [2, 3]. However, during the reperfusion of the donor liver, there were often intraoperative severe electrolyte and acid-base disturbances as well as subsequent hemodynamic instability.

Postreperfusion syndrome (PRS) was initially presented by Aggarwal, defined as a 30% decrease in mean arterial pressure (MAP) from the baseline lasting more than 1 min within the first 5 min after reperfusion of the graft [4]. In particular, PRS was a serious intraoperative complication of liver transplantation that could influence recipient’s morbidity and mortality, and was proved to be an independent risk factor for post-transplantation mortality and occurrence of primary graft non-function [5, 6]. The cause of these severe hemodynamic changes was complex. The important roles in this phenomenon were attributed to the efflux of cold, acidic blood, and electrolyte disturbances, especially hyperkaliemia [7, 8]. Inflammatory cytokines such as interleukin 1 beta (IL-1β) or tumor necrosis factor alpha (TNF-α) produced during ischemia were also possibly responsible for PRS at this time [9]. Different surgical techniques might be considered to prevent the development of PRS by reducing these substances or components into the circulation.

Many previous studies have reported that retrograde reperfusion through inferior vena cava (IVC) could reduce hemodynamic fluctuations, and the reperfusion method had been improved and used in many centers [10–12]. To date, however, it was still unknown from available literature whether this technique led to the better outcome following transplantation [13, 14]. The changes were analyzed in blood gas results and the fluctuations in the arterial pressure during reperfusion of the donor liver using a new type of retrograde reperfusion. In short, this study was designed to investigate the effect of retrograde reperfusion on the patient’s intraoperative internal environment and hemodynamics during classic OLT.

## Methods

Ethical approval number:2015PHB102–01.

### Subjects

After previously approved by the institutional review board of Peking University People’s Hospital, and written, informed consent by all patients, 33 consecutive recipients of OLT between August 2015 and December 2016 were initially considered for inclusion. Recipients with incomplete or unreliable data due to technical difficulties or instability during operation were excluded, leaving a cohort of 30 subjects.

### Donor data

Since the brain death law has not yet been established, and most people cannot accept organ donation in the state of heart beating, organ donation can only be based on heart death as a unified standard in our country. Therefore, all of the liver grafts were used from donation after cardiac death (DCD) donors, including 18 severe brain injuries and 12 cerebrovascular accidents. No donor livers were procured from executed prisoners. The liver donation of each case conformed strictly to the regulation of the Ethical Committee in our hospital. The donors were generally younger than 50 years with body mass index (BMI) lower than 30 kg/m^2^, and serum aminotransferase levels at procurement were less than twice of the normal values. Tests for infectious diseases such as hepatitis B, hepatitis C, acquired immunodeficiency syndrome (AIDS), and syphilis were all negative. Artificial life supports of all DCD donors had a planned withdrawal in the operating room or ICU. An independent physician from the donor hospital was assigned to declare death. Following a mandatory waiting period after asystole, procurement began. Dual perfusion via cannulation of the abdominal aorta and superior mesenteric vein using University of Wisconsin (UW) solution at 4 °C was employed to rapidly and simultaneously reach both the liver and kidney. All the organs were then preserved in 4 °C UW solution.

### Surgical procedures

All 30 patients underwent classic OLT with nonvenovenous bypass (Non-VVB). The detailed surgical procedure using this new type of retrograde reperfusion was as follows: (1) The suprahepatic and infrahepatic IVC were continuously anastomosed using 4–0 Prolene sutures. In anastomosing the infrahepatic IVC, 1000 mL of 2% albumin was used through the portal vein (PV) of the liver graft to flush out the preservation solution. (2) The posterior wall of the PV was continuously anastomosed using 5–0 Prolene suture. (3) The infrahepatic IVC was declamped, whereas the suprahepatic IVC remained to be clamped. The blood from the IVC then perfused the liver graft retrogradely. After 200 mL of blood venting via the PV of the graft, it was clamped. The suprahepatic IVC was then declamped to relieve obstruction of the IVC and partially restore the systemic circulation. (4) 100 mL of blood venting was via the PV of the recipient. (5) The anastomosis of the anterior wall of the PV was performed and then the PV was declamped to establish normal circulation perfusion of the graft. (6) Finally, the hepatic artery and the bile duct were successively anastomosed and declamped.

### Observational indexes

All patients’ blood samples were obtained using 10-mL needles. Blood sampling was done at different time points including: Before blood venting via the portal vein (PV) of the donor liver, 10 mL of blood was collected from the inferior vena cava (T_0_). During retrograde reperfusion through the IVC, 10 mL of blood was collected when the volume of blood venting reached 10 mL (T_1_), 100 mL (T_2_), and 200 mL (T_3_), respectively. Each blood sample was mixed with unfractionated heparin for anticoagulation. 5 mL of blood was analyzed using a NOVA-f–type Blood Gas Analyzer. The remaining 5 mL was centrifugated at 2000 rpm for 10 min in a cooled centrifuge at 4 °C. Then the supernatant plasma was removed within 30 min after collection and stored at − 80 °C until analysis. The enzyme-linked immunosobent assay was performed to determine the concentrations of IL-1β.

### Statistical analysis

SPSS16.0 software package was utilized for statistical analysis. As the Gaussian quantitative variables, blood gas results were expressed as average standard deviation. Because cytokine levels showed a strong skewness of distribution, and were summarized as median (25th–75th percentile). All data were analyzed by repeated measures ANOVA, with multiple comparisons using Bonferroni correction tests. *P* < 0.05 or *P* < 0.01 was defined to be statistically significant.

## Results

### General characteristics

The general characteristics of all 30 patients enrolled in the study were shown in Table 1. The number of primary liver cancer, posthepatitic cirrhosis, alcoholic cirrhosis, liver failure, and primary biliary cirrhosis were 17, 6, 3, 3, and 1, respectively. Patients with primary liver cancer were the most, and other patients were too few to be able to be grouped for statistical analysis. The average of model for end-stage liver disease (MELD), cold ischemic time (CIT) and warm ischemia time (WIT) were 17, 8.2 h and 5.5 min.

**Table 1:** General characteristics of OLT recipients

Age (years)	56 (48–63)
Male/female (n)	21/9
Recipient BMI (kg/m^2^)	22.3 (16.4–27.7)
Primary liver cancer (n)	17
Posthepatitic cirrhosis (n)	6
Alcoholic cirrhosis (n)	3
Liver failure (n)	3
Primary biliary cirrhosis (n)	1
Child A/B/C (n)	14/11/5
MELD score	17 (11–21)
CIT (hours)	8.2 (6.7–11.5)
WIT (minutes)	5.5 (3.5–8.5)

The results were expressed as n or median (25–75 interquartile range)

*BMI* Body Mass Index, *MELD* Model for end-stage liver disease, *CIT* Cold ischemic time, *WIT* Warm ischemia time

### Intraoperative characteristics of OLT recipients

The MAP were 72 and 96 mmHg at before IVC declamped and before portal vein (PV) declamped. PRS only recipients occurred in 6 patients (20%). Length of surgery and anhepatic duration were 473 and 90 min. All operations were completed successfully (Table 2).

**Table 2:** Intraoperative characteristics of OLT recipients

MAP before IVC declamped (mmHg)	72 (67–78)
MAP before PV declamped (mmHg)	96 (86–110)
PRS (n)	6 (20%)
Length of surgery (minutes)	473 (385–571)
Anhepatic duration (minutes)	90 (75–112)

The results are expressed as n or median (25–75 interquartile range)

*MAP* Mean arterial pressure, *IVC* Inferior vena cava, *PV* Portal vein, *PRS* Postreperfusion syndrome

### Blood data at different time points during blood venting

**Fig. 1:**
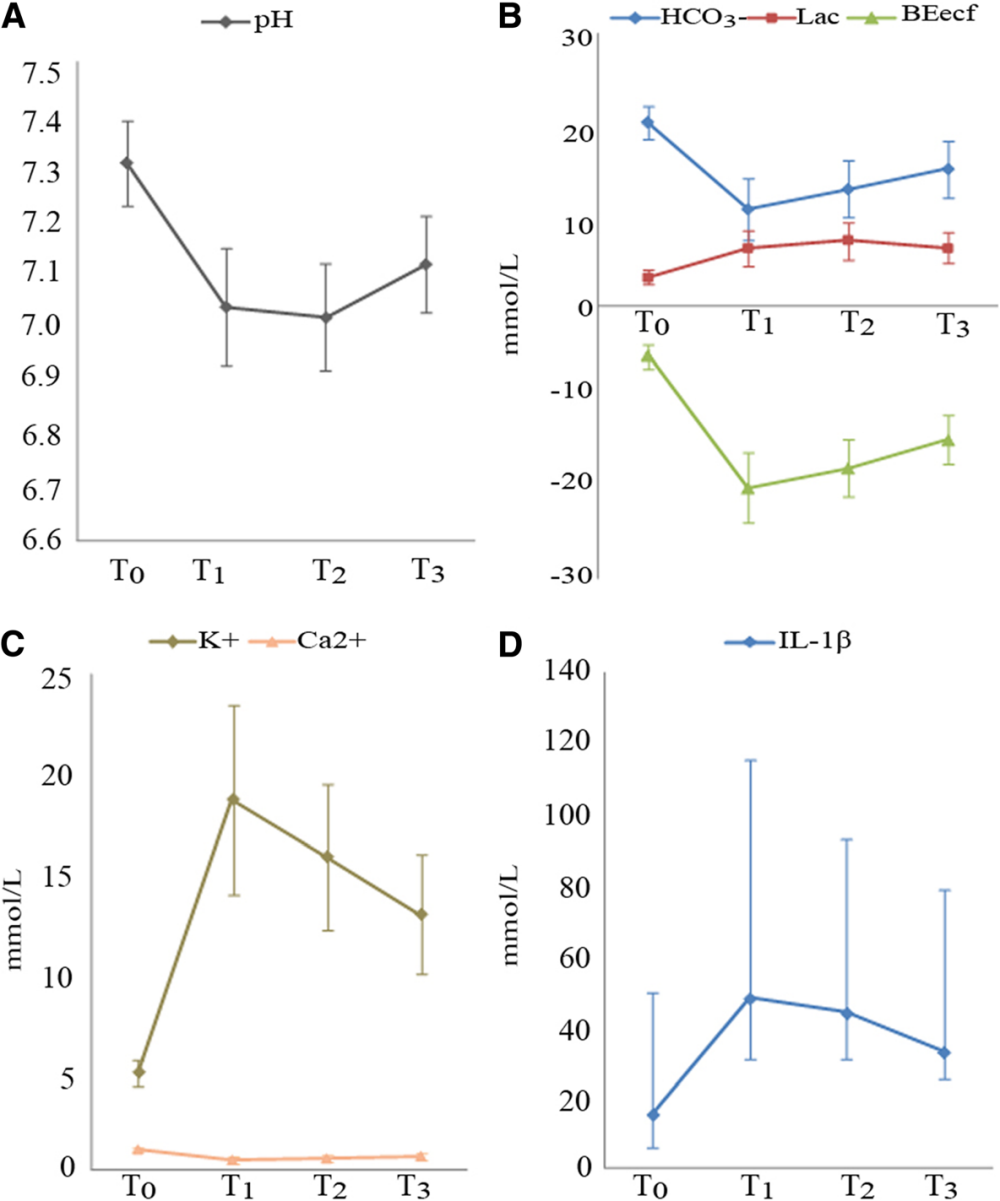
The values of different indexes at different time points. **a** The pH value; **b** The levels of PvO_2_, SvO_2_, and HCO_3_^−^; **c** The levels of Ca^2+^ and K^+^; **d** The level of IL-1β

The blood gas results and cytokines levels at different time points during reperfusion and their evolutions were shown in Table 3 and Fig. 1. The values of pH, PvO_2_, SvO_2_, HCO_3_^−^, BEecf, and Ca^2+^ at T_0_ were higher than T_1_, T_2_ and T_3_ (*P* < 0.05), while values of PvCO_2_, Lac, K^+^, and IL-1β at T_0_ were significant lower than T_1_, T_2_ and T_3_ (*P* < 0.05)_._The most notable findings were the significant changes of nearly all data at T_1_, T_2_ and T_3_, compared with T_0_ and a trend toward recovery in most of the data. Yet their levels at T_3_ did not come back to the levels at T_0_.

**Table 3:** Blood data at different time points during blood venting

Indexes	Each time point during venting				*F* value
	T_0_	T_1_	T_2_	T_3_	
pH	7.31 ± 0.08	7.04 ± 0.11^a^	7.02 ± 0.10^a^	7.12 ± 0.09^abc^	200.303*
PvCO_2_ (mmHg)	35.6 ± 11.3	43.9 ± 16.9^a^	47.6 ± 17.6^a^	55.1 ± 15.1^ab^	135.457*
PvO_2_ (mmHg)	62.1 ± 21.8	56.7 ± 16.4	43.8 ± 12.6^ab^	47.5 ± 13.8^abc^	9.029*
SvO_2_ (%)	78.1 ± 13.2	67.1 ± 15.3^a^	50.3 ± 14.3^ab^	57.7 ± 14.6^abc^	15.892*
Lac (mmol/L)	3.2 ± 0.8	6.4 ± 2.0^a^	7.2 ± 2.0^ab^	6.4 ± 1.7^ac^	57.612*
HCO_3_^−^ (mmol/L)	20.2 ± 1.8	10.7 ± 3.4^a^	12.9 ± 3.1^ab^	15.1 ± 3.1^abc^	125.399*
BEecf (mmol/L)	−5.4 ± 1.6	−20.0 ± 3.9^a^	−17.8 ± 3.2^ab^	−14.7 ± 2.7^abc^	238.295*
K^+^ (mmol/L)	4.9 ± 0.7	18.7 ± 4.8^a^	15.8 ± 3.7^ab^	12.9 ± 3.0^abc^	179.260*
Ca^2+^ (mmol/L)	1.06 ± 0.11	0.49 ± 0.16^a^	0.57 ± 0.17^ab^	0.70 ± 0.16^abc^	124.070*
IL-1β (pg/mL)	15.2 (5.7–49.6)	47.9 (30.6–115.3)^a^	43.7 (30.9–93.2)^a^	32.7 (25.2–78.6)^abc^	41.021*

*P < 0.01

^a^Comparisons from T_1_ to T_0_, T_2_ to T_0_, and T_3_ to T_0_, *P* < 0.05 or *P* < 0.01

^b^Comparisons from T_2_ to T_1_, and T_3_ to T1, *P* < 0.05

^c^Comparisons between T_3_ and T_2_, *P* < 0.05

## Discussion

The method of the donor liver reperfusion had been a constant focus in the research on OLT. As the PV supplied most of liver blood and was easier to be anastomosed than the hepatic artery, initial PV reperfusion anterogradely was the most widely used reperfusion technique [15]. After the suprahepatic IVC, infrahepatic IVC, and PV were conventionally anastomosed, the PV was declamped. The PV blood was reperfused the liver, and blood was vented from the infrahepatic IVC anastomosis. Then the suprahepatic IVC and infrahepatic IVC were declamped successively. The hepatic artery was anastomosed and declamped last. But blood venting was difficult to operate or control due to the deep IVC, and it may be dangerous especially when the exposure was poor. Besides, after recirculation of the liver, a large number of blood stasised in the PV and IVC flowed back into the heart, probably resulting in sharp hemodynamic changes.

Retrograde reperfusion, originated from Kniepeiss at el. [10], was considered to reduce hemodynamic fluctuations.

In this method, it was observed that the PV blood flow was slow, which probably led to inadequate reperfusion of the graft. Most of the IVC blood backflow into the heart might be implicated. Therefore, our study had made some exploration and improvement. Compared with the aforementioned retrograde reperfusion, the suprahepatic IVC remained clamped when the infrahepatic IVC was declamped to allow retrograde reperfusion of the graft in our method. Without the “shunt” or “siphonic” effect due to the declamping of the suprahepatic IVC, the pressure of IVC blood was fully released into the graft to reperfuse it completely. Actually, a large number of cold preservation fluid was viewed and blood was flowed out more quickly during blood venting. Consequently harmful metabolites could be removed more effectively, and the influence of venous return from the graft could be reduced. Nevertheless, some studies found that low-pressure perfusion with low oxygenated blood from the IVC could reduce the production of free oxygen radicals, and IRI of the graft [16, 17]. So further experimental research on the reperfusion method were needed.

As shown in Table 3 and Fig. 1, large amounts of acidic metabolites such as lactate had accumulated in the storage of the liver graft. During reperfusion through the IVC, the cold ischemic and hypometabolic liver graft, restarted its metabolism, and O_2_ was gradually consumed. With the reperfusion going on, most indices of acid-base balance showed a trend toward restoration, especially HCO_3_^−^ and BEecf levels. The results showed that retrograde reperfusion through IVC could eliminate part of the acidic metabolites and slightly reduce patients’ risk of acidosis.

In this study, the levels of K^+^ andCa^2+^ were changed significantly after IVC blood flowed through the donor liver. Hyperkalemia might be due to the residue of potassium in the old preservation solution (K^+^ concentration was 125 mmol/L in the UW solution). The transfer of K^+^ out from within the cells, due to academia, might also be an explanation [18]. Hypocalcemia might be related to the dilution of UW solution. The reduced metabolism of citric acid inside the donor liver was preserved at a low temperature could further lower the lever of Ca^2+^. Remarkably, both hyperkalemia and hypocalcemia were widely considered as the important risk factors for PRS [7, 19, 20], so correction of K^+^ and Ca^2+^ levels after the restoration of the graft’s circulation during classic OLT was essential. Likewise, as mediators of various responses, such as hemodynamic and biochemical alterations, immune system activation, or graft rejection [21], cytokines (IL-1β) were greatly increased during graft reperfusion. It had been previously considered that the liver graft itself could possibly accumulate substantial cytokines [22]. Our results showed that the liver graft indeed included significantly higher concentrations of cytokines in comparison with those found in the patient’s systemic circulation, which had been directly demonstrated only in few studies until now [23, 24].

All the levels of K^+^, Ca^2+^ and IL-1β were gradually returned to the levels of T_0_ as blood venting was increased. Although there were still significant differences between the end of blood venting and T_0_, respectively, it could be expected that continued blood venting would decrease the K^+^, IL-1β levels and increase Ca^2+^ level further or might even help to restore them to the levels of T_0_. However, patients with more than 250 mL of blood venting would have greater hemodynamic fluctuations, while 200 mL of blood venting during the surgery would lead to minor hemodynamic fluctuations and have relatively less influence on the operation and patients. As a result, retrograde reperfusion reported in the present study employed 200 mL blood venting via the PV, and was found to effectively reduce electrolyte disorders and remove the excess of cytokines from the graft after recirculation with OLTs.

Several recent studies had indicated that PRS was associated with many factors [20, 25]. Within the cold, high-potassium and acid blood, the donor liver released a lot of vasoactive substances (including IL, TNF, cystatin and other cytokines, activated complement segments, and NO) back into the heart [26]. At the same time, the recipient reactively released large amounts of cytokines (such as IL-6, IL-8, CCL-1 and CD40 ligands) and other vasoactive substances [27]. By activating the kallikrein kinin system (KKS) and releasing bradykinin, they reduced systemic resistance, resulting in decreased arterial pressure.

According to the diagnostic criteria of PRS suggested by Aggarwal [4], the incidence of PRS during classic OLTs using conventional reperfusion was as high as 30%, while the incidence of PRS was relatively low (20%) in the present study, in which the MAP value before PV declamped was used as the baseline. Because MAP of patients increased after IVC declamped, the incidence of PRS would be lower (3/30) if we used the MAP value before IVC was declamped as the baseline. The possible reasons might be the fact that this new type of retrograde reperfusion effectively removed harmful metabolites from the graft [28]. Also, the IVC blood with a relatively low oxygen content led to low-pressure perfusion of the graft and thus avoided a massive outbreak of oxygen radicals [17]. This maneuver also helped to decrease the incidence of PRS and to some extent reduced the severe hemodynamic fluctuations.

## Conclusions

In conclusion, retrograde reperfusion could eliminate some of the harmful metabolites inside the donor liver in time and to some extent reduce acid-base and electrolyte disorders as well as drastic hemodynamic fluctuations after recirculation.

## Abbreviations

AIDS: Acquired immunodeficiency syndrome
CIT: Cold ischemic time
IL-1β: Interleukin 1 beta
IVC: Inferior vena cava
KKS: Kallikrein kinin system
MAP: Mean arterial pressure
MELD: Model for end-stage liver disease
Non-VVB: Nonvenovenous bypass
OLT: Orthotopic liver transplantation
PRS: Postreperfusion syndrome
PV: Portal vein
TNF-α: Tumor necrosis factor alpha
UW: University of Wisconsin
WIT: Warm ischemia time
